# A CRISPR Screen Identifies the E3 Ubiquitin Ligase Rfwd2 as a Negative Regulator of Glucose Uptake in Brown Adipocytes

**DOI:** 10.3390/genes14101865

**Published:** 2023-09-26

**Authors:** Matthew D. Lynes, Qian Huang, Carolina Cora, Sheng-Chiang Su, Peng Yi, Yu-Hua Tseng

**Affiliations:** 1 Center for Molecular Medicine, Maine Health Institute for Research, Scarborough, ME 04074, USA; 2 Department of Medicine, Maine Health, Portland, ME 04101, USA; 3 Graduate School of Biomedical Science and Engineering, University of Maine, Orono, ME 04469, USA; 4 Roux Institute at Northeastern University, Portland, ME 04101, USA; 5 Section on Integrative Physiology and Metabolism, Joslin Diabetes Center, Harvard Medical School, Boston, MA 02215, USA; qhuang08@nyit.edu (Q.H.); doc10504@gmail.com (S.-C.S.);; 6 Division of Endocrinology and Metabolism, Department of Internal Medicine, Tri-Service General Hospital, National Defense Medical Center, Taipei 114, Taiwan; 7 Harvard Stem Cell Institute, Harvard University, Cambridge, MA 02138, USA

**Keywords:** brown adipose, adipocyte, glucose

## Abstract

Brown adipose tissue activation increases energy expenditure and has been shown to improve glucose tolerance, making it a promising target for the treatment of obesity and type 2 diabetes. Brown adipocytes differentiate into cells with multilocular lipid droplets, which can efficiently absorb and oxidize glucose; however, the mechanisms regulating these processes are not completely understood. We conducted a genome-wide loss-of-function screen using a CRISPR-based approach to identify genes that promote or inhibit adipogenesis and glucose uptake in brown adipocytes. We validated genes that negatively or positively regulated these pathways and verified that the E3-ubiquitin ligase Rfwd2 suppressed brown adipocyte glucose uptake. Brown adipocytes with CRISPR-targeted Rfwd2 deletion showed an altered proteomic landscape and increased basal, as well as insulin-stimulated, glucose uptake. These data reveal the complexity of genetic regulation of brown adipogenesis and glucose metabolism.

## 1. Introduction

Lipid and glucose metabolism are closely interconnected, especially in adipose tissue, and maintaining a healthy balance between the two is essential for homeostasis. Glucose in the bloodstream is transported to cells throughout the body, where it is taken up and metabolized to produce energy or converted into other forms for storage [1]. One cell type that can contribute to systemic glucose disposal is the brown adipocyte, where glucose from the blood can be oxidized in cells or converted into lipids via de novo lipogenesis [2]. In humans, glucose uptake is currently the gold standard means of measuring brown adipose tissue activity [3,4,5]. Clinical studies of the effects of cold temperature, as well as adrenergic agonists, have shown that brown adipocyte activation increases energy expenditure by stimulating glucose uptake and disposal [6,7,8]. The accumulation of excess fat in the body can have a significant impact on metabolism, leading to insulin resistance and impaired glucose uptake. Therefore, maintaining a balance between fat storage and glucose levels is essential. Consistent with its impact on systemic metabolism, brown fat glucose uptake is negatively associated with body mass index (BMI), suggesting that increasing brown fat glucose uptake could decrease BMI [9,10]. Additionally, because brown adipocytes can expend stored energy through non-shivering thermogenesis in response to cold temperature [11], brown fat can act as a glucose sink, making therapeutic strategies that increase brown fat activity an attractive target for combating obesity and diabetes [1].

The complex genetic system regulating the cellular enzymes, signaling pathways, and hormones that underlie brown adipocyte activation are critical to the biological basis for this tissue in contributing to whole body metabolism [12]. A recent study highlighted the role of insulin signaling in mediating brown adipose tissue glucose and lipid uptake, with adrenergic stimulation of lipolysis playing a critical role in insulin release [13]. Although adrenergic and insulin signaling are known to be major components of this network, it is possible that other unidentified genes form a constituent of brown adipocyte glucose uptake. Both adrenergic and insulin receptors are expressed in several different tissue types, meaning neither can fully explain the tissue-specific activation of brown adipocyte glucose uptake that occurs during cold exposure, which suggests other pathways may play a role [11,12].

CRISPR/Cas9-based methods have facilitated new strategies for genome-wide, loss-of-function screens, to elucidate genetic regulators of cellular function in cultured cells [13,14]. Using CRISPR screens in HeLa cells, the genetic circuitry of exocytosis was scanned, identifying Rab-interacting factor (RABIF) as a key component of insulin-regulated glucose transport [15]. However, no CRISPR screens using brown adipocytes have been previously reported. To directly unravel the positive and negative regulators of glucose uptake in brown adipocytes, we applied a CRISPR screen to systematically measure the effects of genetic targeting on the uptake of a fluorescence-based glucose reporter dye. Our screen was able to capture the distribution of more than 78,000 gRNAs in brown adipocytes stained for glucose uptake, determining the key genetic circuitry required for adipogenesis. By validating candidate gRNAs, we identified the E3 ubiquitin ligase Rfwd2 as a mediator of brown adipocyte glucose uptake.

## 2. Materials and Methods

### 2.1. Cell Culture

DE cells were originally isolated from the interscapular brown adipose tissue of newborn C57BL/6 mice and immortalized by infection with a retrovirus expressing SV40 T antigen, followed by selection with G418 [16]. DE cells were cultured in high-glucose DMEM with 10% FBS Dulbecco’s modified Eagle’s medium (high glucose) containing 10% bovine serum at 37 °C in a 5% CO_2_ incubator. Adipogenic differentiation was induced using a published protocol; briefly, cells were grown to 90% confluence and induced with the addition of 20 nM insulin along with 1 nM T328 to culture media for two days. Cells were then differentiated by adding 20 nM insulin, 1 nM T3, 0.5 mM IMBX, 0.5 μM Dexamethasone, and 125 mM Indomethacin for 7 days, with media change every other day. Cells were considered mature 9 days after induction and kept in complete media for one day before experiments. Mature adipocytes were used at day 9 and kept in serum-free DMEM throughout the study period.

### 2.2. Viral Transduction

DE cells cultured in 25 cm plates were infected with the Brie CRISPR Library (Addgene #73633) Brie library lentiviral production, titer, and transduction were performed as previously described [17]. We infected twelve 15 cm plates at an MOI = 0.3 with the Brie library in the lentiCRISPRv2 backbone in the presence of 10 mg/mL polybrene, and 48 h after infection we used puromycin (2 μg/mL) to select for a further 72 h. Cells were then cultured in DMEM containing 10% bovine serum, until 90% confluency. At this point, two plates were lysed for the preadipocyte samples and genomic DNA (gDNA) was extracted from cells using a Quick-gDNA Midiprep kit (Zymo Research, Irvine, CA, USA) and subjected to NGS, to determine sgRNA distribution. The other 10 plates were adipogenically differentiated, as previously described. After differentiation, cells were counted and two plates were lysed for the adipocyte samples and gDNA was extracted, with the final eight plates used for 2-NBDG staining and sorting.

To generate individual gene knockouts, gRNAs were cloned into the lentiCRISPRv2 backbone and DE preadipocytes were infected in 10 cm cell culture dishes using the same protocol that was used for the initial screen. To generate Rfwd2 KO cells, two gRNAs (gRNA1: CGAGCGTGTAGTTCTCCGAG gRNA2: ACTGGGATCCTTAGGCAAAG) were designed and cloned into the PX459 backbone for transfection. A 1:1 mixture of the two plasmids was used for lipofectamine transfection (Thermo Fisher, Waltham, MA, USA), after which culture, puromycin selection, and adipogenic differentiation were performed as previously described.

### 2.3. 2-NBDG Staining and Sorting

For 2-NBDG staining and sorting, differentiated DE brown adipocytes carrying the Brie CRISPR library were stained with 2-NBDG (Thermo Fisher, Waltham, MA, USA) according to the manufacturer’s directions. Briefly, on the morning of staining the induction media were removed and replaced with fresh DMEM. Cells were treated with 100 nM insulin for 15 min, before the addition of 100 uM 2-NBDG. After 1 h incubation at 37 °C in a 5% CO_2_ incubator, cells were detached from the plate with Trypsin/EDTA and sorted on a FACSARIA 2 FACS machine (BD Biosciences, San Jose, CA, USA). Just before sorting, propidium iodide was added to the cells at a final concentration of 1 ng/mL (Thermo Fisher, Waltham, MA, USA). All plots were made using FlowJo software (Version 10.8, FlowJo LLC). We sorted approximately 5 × 10^6^ cells from each of the 8 plates to achieve the recommended representation of ~400 cells per sgRNA (4 × 10^7^ total cells sorted). After sorting, gDNA was extracted as previously described. Illumina sequencing was performed by a commercial vendor (Novogene, Nanjing, China). For sorted cells, we acquired a total of 2.98 × 10^8^-reads from 4 × 10^7^ total cells, for approximately 7× read coverage. For preadipocytes and adipocytes, we acquired a total of 1.08 × 10^8^-reads from 3 × 10^7^ total cells, for approximately 3× read coverage.

### 2.4. Oil Red O Staining

Cells were washed twice with PBS and fixed with 10% buffered formalin for 30 min at room temperature. Cells were then stained with a filtered Oil Red O solution (0.5% Oil Red O in isopropyl alcohol) for 2 h at room temperature and washed several times with distilled water before visualization.

### 2.5. 2-DOG Uptake

Cells were serum starved in no-glucose DMEM (Gibco; catalog# 11966025, Thermo Fisher, Waltham, MA, USA) for 4 h before the treatments. The cells were treated with PBS or insulin (1 uM, Sigma Aldrich Cat# I9278, Sigma Aldrich, St. Louis, MO, USA) for 30 min before starting the glucose transport procedure. Cells were washed once with HEPES Buffered Saline (HBS), which was subsequently completely aspirated. After that, 300 μL of transport solution (TS) containing [3H]2-deoxyglucose (0.5 uCi/mL) and 2-deoxyglucose (5 mM) diluted in 20 mM HBS solution was added. Cells were incubated in this solution for 5 min at room temperature, which was then quickly aspirated. An ice-cold stop solution (0.9% Saline) was added and washed, before the addition of 0.5 mL of a 0.05 M NaOH solution to the wells. Cells were scraped and homogenized, and the homogenate was transferred to fresh scintillation tubes (0.35 mL), where it was vigorously mixed with 4 mL of liquid scintillation cocktail (CytoScint™-ES Liquid Scintillation Cocktail, MP Biomedicals, Santa Ana, CA, USA) prior to scintillation counting. Protein levels were determined by BCA assay in the remaining homogenate for the normalization of values.

### 2.6. PCR

PCR genotyping primers were designed using Primer3 primer design software (Version 4.1.0). Genomic DNA was isolated as described previously, PCR was performed with a PlatinumTaq Green Kit (Thermo Fisher, Waltham, MA, USA) and electrophoresed on a standard agarose gel, with ethidium bromide added for detection.

### 2.7. Immunoblotting

Cells were differentiated into mature brown adipocytes according to a standard adipogenic differentiation protocol. After that, cells were scraped from tissue-culture plates into RIPA buffer (Boston BioProducts Inc., Ashland, MA, USA) supplemented with protease and proteinase inhibitors cocktails (Sigma-Aldrich, Dallas, TX, USA) and further homogenized for protein detection. Protein concentrations were determined using a Pierce BCA kit (Life Technologies, Carlsbad, CA, USA), according to the manufacturer’s instructions. For immunoblots, lysates were diluted into Laemmli buffer and boiled for loading onto 10% Tris gels for SDS–PAGE. After complete separation of the proteins, they were transferred onto a PVDF membrane (Amersham Biosciences, Amersham, UK), blocked in western blocking buffer (Roche, Basel, Switzerland), and primary antibodies were applied in blocking buffer overnight at 4 °C. After washing 4× for 15 min with TBS-T, secondary antibodies were applied for 1 h in blocking buffer. Membranes were washed again 3× times for 15 min in TBS-T and developed using chemiluminescence (Thermo Fisher, Waltham, MA, USA).

### 2.8. Proteomics

For proteomic analysis of WT and Rfwd2 KO brown adipocytes, cells were lysed in buffer (50 mM Tris-HCl, pH 7.5, 150 mM NaCl, 2 mM EDTA, 1% NP-40, 10% glycerol, 1% Sodium Deoxycholate) containing protease inhibitor cocktail, PR-619, and MG-132 on ice for 15 min. Lysates were cleared using high-speed centrifugation (~14,000× *g*) for 10 min at 4 °C. The supernatants were collected, and the protein concentration of each supernatant was determined using a BCA Protein Assay (Pierce). Trypsin digestion and LC-MS/MS analysis was performed by a commercial vendor (LifeSensors Inc., Devault, PA, USA). MS data were searched against the UniProt mouse database (10 January 2018) using MaxQuant 1.6.2.3. The protein false discovery rate was set at 1%. As larger proteins generate more peptides, the intensity values were adjusted by normalizing against the number of theoretical peptides for each protein (iBAQ intensity).

### 2.9. Data Analysis and Statistics

The differential gRNA abundance was calculated first by filtering genes with less than 1 count per million (CPM) in at least half of the group size (4 samples). Counts were normalized using weighted trimmed mean of M-values (TMM), and voom transformation with quality weights was used for PCA. We used limma, an R package that powers differential expression analyses, with samples weighted and adjusted for the effects of technical replicate plates. For pathway analysis, we used rotation gene set test using weighted samples and adjusted for the effects of technical replicate plates. gRNA normalized distributions and comparisons can be found in Supplementary Table S1.

No statistical method was used to predetermine the sample size. The experiments were not blinded. All statistics were calculated using R Studio (Version 2023.03.1+446), Microsoft Excel (Version 16.77), and GraphPad Prism (Version 9). An unpaired Student’s *t*-test was used to compare only 2 groups, and one-way ANOVA followed by a Tukey’s post hoc test was conducted when comparing more than 3 groups. *p* < 0.05 was adopted as significant, unless otherwise specified.

## 3. Results

### 3.1. Generation of a Mature Brown Adipocyte Cell Library

**Figure 1:**
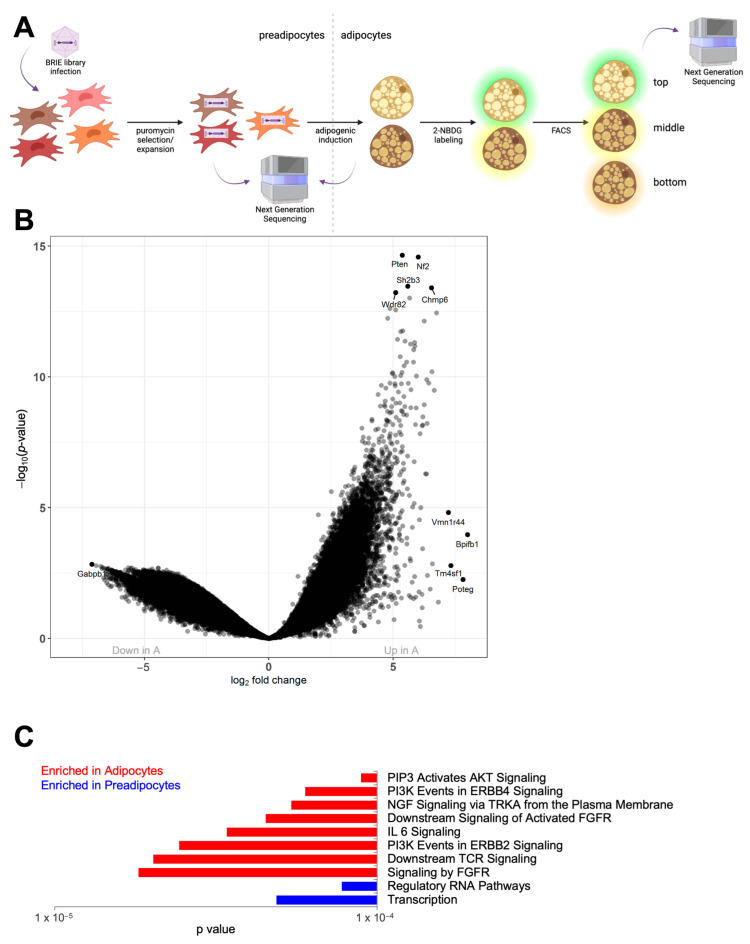
**Generation of a mature brown adipocyte cell library**. (**A**) Schematic of the screen, in which murine brown preadipocytes were infected with the BRIE CRISPR knockout library, selected, expanded, differentiated, stained with 2-NBDG, and sorted into different populations based on glucose uptake. Samples were taken as indicated for next generation sequencing of the gRNAs in each population of cells. Image created in BioRender. (**B**) Volcano plot showing the log2 fold change between adipocyte (A) and preadipocyte (P) samples versus the –log10 of the adjusted *p*-value for each individual comparison, with several genes highlighted. N = 2 technical replicate samples per group. (**C**) Comparison of the differentially represented gRNAs using gene ontology (GO) analysis showing the top 10 differentially represented pathways between A and P samples by adjusted *p*-value. For all charts, N = 2 technical replicate samples per group.

In the search for genetic regulators of brown adipocyte glucose uptake, we performed a two-tiered, pooled, genome-wide CRISPR screen for uptake of the fluorescent glucose analog 2-Deoxy-2- [(7-nitro-2,1,3-benzoxadiazol-4-yl)amino]-D-glucose (2-NBDG) on immortalized brown adipocytes in vitro (Figure 1A). To perturb gene function, we used the BRIE lentivirus library, which encodes 78,637 sgRNAs targeting 19,674 annotated protein-coding genes (4 sgRNAs per gene). Viral infection is technically challenging in mature adipocytes; therefore, we infected preadipocytes with the BRIE library prior to adipogenic differentiation. Cells were then stained with 2-NBDG, for glucose uptake measurement with flow cytometry. Since cells were infected before adipogenesis, we began by comparing the gRNAs that were sequenced in preadipocytes (P) to the gRNAs that were sequenced from mature adipocytes (A) to identify putative regulators of adipogenic differentiation (first tier of the screen) (Supplementary Figure S1A–C). Interestingly, one of the most significantly enriched gRNAs found in the A population was the transcript coding for Pten, known for being a major negative regulator of insulin signaling (Figure 1B). Previous studies have shown that Pten promotes brown adipogenesis [18] and deletion of Pten in brown adipocytes precursors decreases their thermogenic potential [19].

The preadipocyte comparison with mature adipocytes identified a large number of differentially expressed gRNAs in these two populations (Supplementary Figure S2A–D, Supplementary Table S1). Furthermore, when examining the differences between preadipocyte and adipocyte gRNA profiles using gene ontology analysis (GO pathways), we discovered pathways that were enriched in the differentiated populations. In mature adipocytes, gRNAs targeting genes related to proadipogenic pathways such as AKT and PI3 Kinase were enriched (Figure 1C). Pathways that were depleted from mature adipocytes (and enriched in the preadipocyte population) were related to basic functions of transcription, consistent with the necessity of these pathways for cell function and survival. Analysis of putative regulators of glucose uptake identified in the second tier of the screen must be taken in the context of the first adipogenic selection applied during the first tier of the screen.

### 3.2. Screening 2-NBDG Uptake in Mature Adipocytes

**Figure 2:**
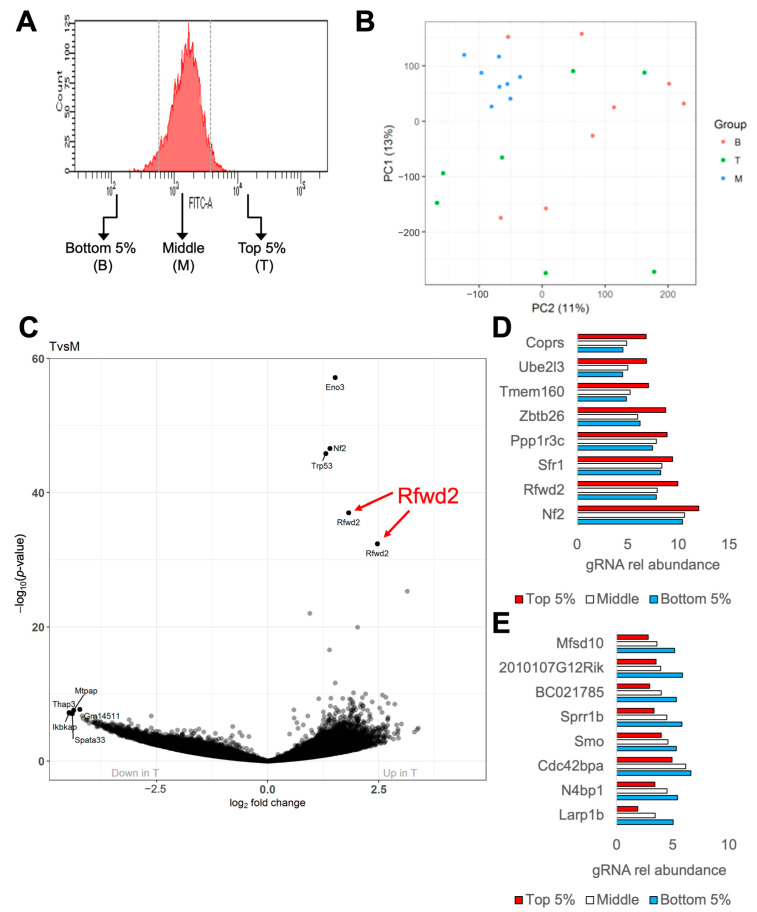
**Screening 2-NBDG uptake in mature adipocytes.** (**A**) Example of sorting on a population of mature adipocytes infected with the BRIE CRISPR library and stained with 2-NBDG, showing sorting of the bottom 5% (B), top 5% (T), and middle (M) samples of cells by fluorescence intensity. (**B**) PCA chart showing B, T, and M populations using the weighted frequency of the gRNAs sequenced from each population. (**C**) Volcano plot showing the log2 fold change between top 5% (T) and middle (M) samples versus the –log10 of the adjusted *p*-value for each individual comparison, with several genes highlighted. N = 8 technical replicate plates per group. (**D**) Top 8 gRNAs from our screen with distributions across the T, M, and B populations that showed a consistent increase representative of negative regulation of glucose uptake, ranking these genes by false discovery rate. (**E**) Top 8 gRNAs from our screen with distributions across the T, M, and B populations that showed a consistent decrease representative of positive regulation of glucose uptake, ranking these genes by false discovery rate.

In the second tier of our screen, we used differentiated adipocytes stained with a fluorescent glucose analog to investigate the genetic pathways that regulate glucose uptake in brown fat cells. We stained mature adipocytes carrying the BRIE library with 2-NBDG and then sorted them based on 2-NBDG fluorescence using flow cytometry (Figure 2A and Figure S3A). We analyzed the gRNA profiles of the brightest top 5% (T), the dimmest bottom 5% (B), and the remainder of the cells (M) (Supplementary Figure S3B,C, Supplementary Table S1). After weighing and adjusting each sample to account for the number of gRNA reads, principal component analysis (PCA) of the gRNA profile showed a clear clustering of the M samples. However, the T and B populations did not form distinct clusters (Figure 2B). To examine putative regulators of brown adipocyte glucose uptake, we began by comparing the abundance of gRNAs in the T population to their abundance in the M population using a volcano plot (Figure 2C). We found several gRNAs that were enriched in the cells with the highest glucose uptake, including two gRNAs for the gene Rfwd2, as well as gRNAs for Nf2, Eno3, and Trp53. To further refine the list of putative genetic regulators of brown adipocyte glucose uptake, we explored the gRNA distribution across the T, M, and B populations that showed either a consistent increase representative of negative regulation of glucose uptake (Figure 2D) or a consistent decrease indicative of positive regulation of glucose uptake (Figure 2E), and we ranked these genes by their false discovery rate. The top eight positive regulators and the top eight negative regulators of glucose uptake were tested in subsequent validation studies.

### 3.3. Validating Putative Genetic Regulators of 2-NBDG Uptake

**Figure 3:**
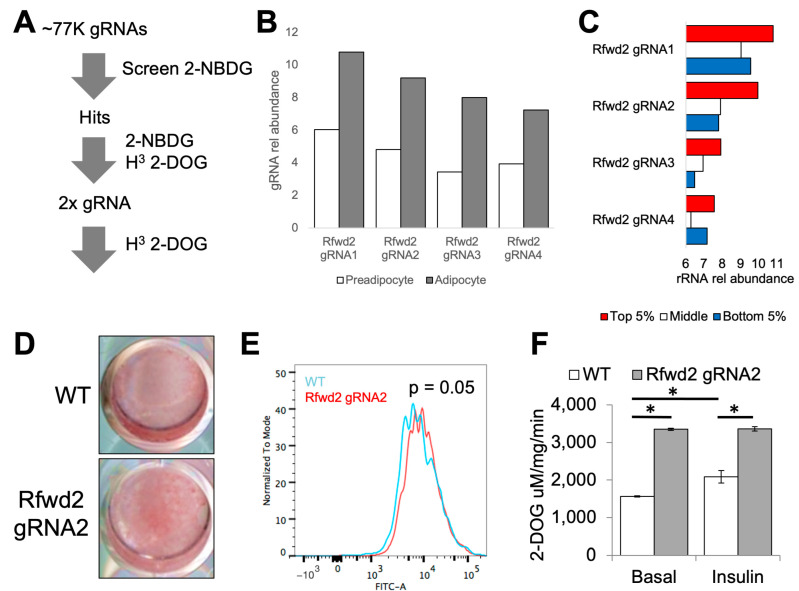
**Validating putative genetic regulators of 2-NBDG uptake.** (**A**) Schematic of validation pipeline for first validating the specific gRNAs that were identified in the initial screen, followed by follow-up targeting of validated loci. (**B**) Rfwd2 gRNA relative abundance in preadipocytes compared to differentiated adipocytes. (**C**) Rfwd2 gRNA relative abundance in 2-NBDG-sorted brown adipocytes. (**D**) Oil red-O-stained lipid accumulation in adipogenically differentiated wild-type brown adipocytes and cells edited with Rfwd2 gRNA2. (**E**) Histogram of 2-NBDG uptake measured by flow cytometry in adipogenically differentiated wild-type brown adipocytes and cells edited with Rfwd2 gRNA2. The *p*-value shown was calculated by Chi-square test. (**F**) Basal and insulin-stimulated tritiated 2-DOG uptake in adipogenically differentiated wild-type brown adipocytes and cells edited with Rfwd2 gRNA2. Data are shown as the mean value of 3 technical replicates, * indicates *p* value less than 0.05 using Students *t*-test.

To validate the putative genetic regulators of brown adipocyte glucose uptake that were identified in this study, we designed a pipeline to measure 2-NBDG uptake in addition to tritiated 2-deoxyglucose (H3 2-DOG) uptake in cells with a targeted disruption of the genes identified in the screen (Figure 3A). To generate loss-of-function cells for each gene, we used both the original gRNAs that were described in the screen, as well as a second strategy employing two different gRNAs that target a single gene in combination. Of the 16 targets that were identified for validation, we selected the gene Rfwd2, which codes for the synonymous E3 ubiquitin-protein ligase Rfwd2 (also known as Cop1), for further study based on several criteria. First, all of the gRNAs targeting Rfwd2 in our screen were significantly enriched after differentiation, suggesting Rfwd2 is dispensable for adipogenesis (Figure 3B). Second, all four Rfwd2-specific gRNAs in our screen were found most often in the Top 5% of cells stained with 2-NBDG (Figure 3C). We validated these observations in brown adipocyte cultures transduced with the highest scoring gRNA targeting Rfwd2 identified in our screen (gRNA1) using Oil red O staining (Figure 3D) and 2-NBDG flow cytometry analysis (Figure 3E). Furthermore, we observed increased 2-DOG uptake in both the basal and insulin-stimulated state in mature adipocytes targeted with the Rfwd2 gRNA1 (Figure 3F).

### 3.4. Rfwd2 Targeting Decreases Protein Levels and Increases Glucose Uptake

**Figure 4:**
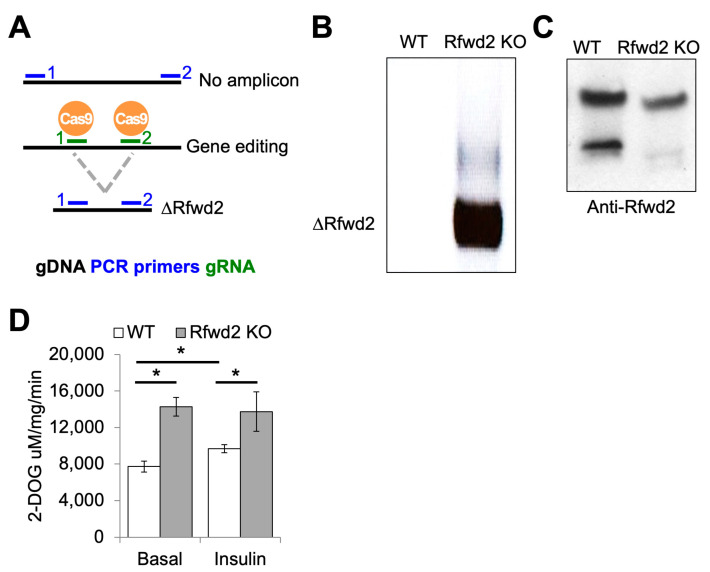
**Rfwd2 targeting decreases protein levels and increases glucose uptake.** (**A**) Schematic of targeting scheme for generation of Rfwd2 KO cells with two different gRNAs (green) and validation of knockout using allele-specific PCR primers (blue). (**B**) Gel electrophoresis of DNA amplified with Rfwd2 allele-specific PCR primers in wild-type and Rfwd2 KO brown adipocytes. (**C**) Immunoblotting for Rfwd2 in wild-type and Rfwd2 KO brown adipocytes. (**D**) Basal and insulin-stimulated tritiated 2-DOG uptake in adipogenically differentiated wild-type brown adipocytes and Rfwd2 KO cells. Data are shown as the mean value of 3 technical replicates, * indicates *p* value less than 0.05 using Students *t*-test.

We wanted to confirm that the effect of the Rfwd2 gRNA1 was caused by Rfwd2 loss of function. We transfected cells with a pair of gRNAs that targeted genomic loci approximately 950 base pairs apart surrounding exon 1 of Rfwd2, generating Rfwd2 KO cells (Figure 4A). PCR analysis of genomic DNA in these cells confirmed the presence of a knockout band that was not present in the parental cell line (Figure 4B). Immunoblotting for Rfwd2 on wild-type and knockout cells showed a decrease in a higher, heavier band that immunoreacted with the Rfwd2 antibody we used, together with the absence of a lower molecular weight band in knockout cells (Figure 4C). Importantly, we validated the effect of CRISPR targeting of Rfwd2 in brown adipocytes on 2-DOG uptake with increased basal and insulin-stimulated glucose uptake in KO cells compared to control cells (Figure 4D).

### 3.5. Identifying Putative Targets of Rfwd2-Mediated Proteolysis in Brown Adipocytes

**Figure 5:**
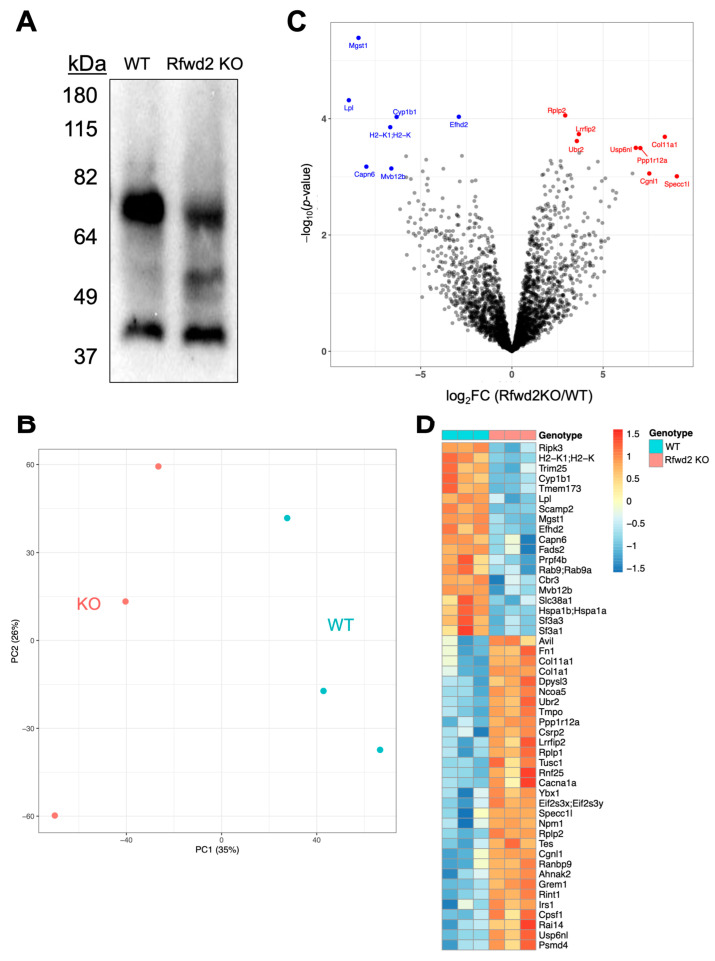
**Identifying putative targets of Rfwd2-mediated proteolysis in brown adipocytes.** (**A**) Immunoblotting for ubiquitinated proteins in wild-type and Rfwd2 KO brown adipocytes. (**B**) PCA of technical replicate samples of wild-type and Rfwd2 KO cell proteomic signatures measured through LC-MS. (**C**) Volcano plot showing the log2 fold change between Rfwd2 KO and WT samples versus the –log10 of the adjusted *p*-value for each individual comparison, with several proteins highlighted. N = 3 technical replicate samples per group. (**D**) Heatmap of proteins detected by LC-MS in WT and Rfwd2 KO brown adipocytes.

Owing to Rfwd2 encoding an E3 ubiquitin ligase, we reasoned that dysregulated proteostasis may have underlain the enhanced glucose uptake we observed in these cells. Consistently with this model, by measuring total ubiquitinated protein in wild-type and Rfwd2 knockout cells by immunoblotting, we found an altered pattern of ubiquitinated proteins consistent with modified ubiquitin ligase function (Figure 5A). To establish putative targets of Rfwd2 that mediate its effect on glucose uptake, we measured the total proteome of Rfwd2 knockout cells through mass-spectrometry and compared it to wild-type cells. In total, we identified 3872 distinct proteins in control and Rfwd2 cells, where PCA analysis of the three technical replicates used for each cell line confirmed a clear separation between the principal components of the proteins in these two cell types (Figure 5B and Figure S3A, Supplementary Table S2). Given the clear separation between wild-type and Rfwd2 knockout cells in principal component 1, we analyzed the top 50 genes by loading score in PC1 and identified the oxidoreductase activity as enriched amongst genes differentially expressed between these cells, consistently with alterations to their redox activity (Supplementary Table S3). Using volcano plot analysis, we observed 498 proteins that were significantly enriched or depleted in the Rfwd2 knockout cells compared to control cells, with a fold change greater than 2 or less than 0.5 and a false discovery rate less than 0.05 (Figure 5C). Heatmap analysis of the top 50 proteins ranked by false discovery rate was used to identify specific proteins that were differentially regulated by Rfwd2, including increased insulin receptor substrate 1 (Irs1) and decreased lipoprotein lipase (Lpl) (Figure 5D).

## 4. Discussion

In search of protein-coding genes involved in the regulation of glucose uptake, we performed a CRISPR knockout screen in brown preadipocytes. We chose this approach because it could identify previously unknown regulators of glucose uptake in a highly relevant cell type. We validated the gene Rfwd2 as a negative regulator of glucose uptake in brown adipocytes, and loss of Rfwd2 caused a change in proteins involved in PI3K signaling that may mediate this effect. Further investigation into the role of Rfwd2 in glucose uptake and brown adipocyte metabolism is warranted.

Rfwd2 encodes a protein also known as constitutive photomorphogenic protein1 (mCOP1), which ubiquitinates different substrates such as C/EBPa [20], p53 [21] and c-Jun [22], depending on the cell line. In the mouse liver, Rfwd2 ubiquitinates ACC and suppresses fatty acid synthesis; however, it is also highly expressed in mouse interscapular brown adipose tissue [23]. Taken together with our new results that show Rfwd2 suppresses glucose uptake in brown adipocytes, targeting this pathway may promote the glucose-scavenging ability of brown adipose tissue. Changes in the proteomic signature, including increased expression of Irs1 in brown adipocytes lacking Rfwd2, could explain this increased insulin-independent glucose uptake phenotype. We also noted decreased expression of Lpl in Rfwd2 knockout cells, which could suggest that decreasing lipolysis increases glucose uptake. In liver cells, however, Rfdw2 knockout actually increases expression of adipose tissue triglyceride lipase (ATGL), so it is unclear what the effect on lipolysis would be in fat cells [24].

Our screen had several limitations, which could limit the conclusions that can be drawn, especially from non-validated genetic targets. First, infecting mature lipid-containing adipocytes to manipulate gene expression is inefficient, due to the low gene silencing ability and cytopathogenicity associated with the adenovirus [25,26,27]; therefore, we introduced the viral encoded CRISPR screening line in preadipocytes before adipogenic differentiation. Using this strategy to detect genes involved in glucose uptake, our analysis method is less sensitive to genes that play a role in 2-NBDG staining if their loss interferes with adipogenic differentiation. Furthermore, it is possible that 2-NBDG staining does not reflect glucose uptake and ultimately glucose metabolism in vitro or in vivo. Nevertheless, this reagent has been used with flow cytometry in other cell types [28,29]. In addition, we validated glucose uptake phenotypes with radiolabeled 2-DOG. The modest effect of insulin on glucose uptake into brown adipocytes in vitro underscores the importance of our efforts to identify new putative regulators of glucose uptake in these cells. Finally, our screen utilized immortalized mouse brown adipocytes and was able to phenotype over 100 individual cells, on average, for each individual gRNA in our screen. 

In addition to being a promising tool for correcting disease-causing genetic variants, CRISPR/Cas9 is a compelling tool for use in search of targets for therapeutic development. This forward genetic approach allowed us to classify Rfwd2 as a negative regulator of glucose uptake in brown adipocytes, where it may link glucose uptake to cellular lipid metabolism.

## Data Availability

The authors declare that the data supporting the findings of this study are available within the paper and its Supplementary information files. Correspondence and requests for materials should be addressed to Matthew D. Lynes or Yua-Hua Tseng.

## Supplementary Materials

The following supporting information can be downloaded at: https://www.mdpi.com/article/10.3390/genes14101865/s1, Supplementary Figure S1. (A) Total sequencing read counts for each sample. (B) Sample weights for each sample. (C) Normalization factor for each sample analyzed in the screen. Supplementary Figure S2. (A) PCA of technical replicate samples of preadipocyte and adipogenically differentiated brown fat cell proteomic signatures measured by LC-MS. (B) FDR histogram of gRNAs differentially expressed in preadipocytes compared to adipogenically differentiated brown adipocytes. (C) Heatmap of the top 50 anti-adipogenic gRNAs enriched in differentiated brown adipocytes. (D). Heatmap of the top 50 pro-adipogenic gRNAs enriched in brown preadipocytes. Supplementary Figure S3. (A) Gating strategy to identify single, live (Propidium Iodide negative) cells and sort by 2-NBDG staining. (B) FDR histogram of gRNAs differentially expressed in the top 5% highest stained brown adipocytes by 2-NBDG compared to the middle population. (C) FDR histogram of gRNAs differentially expressed in the bottom 5% highest stained brown adipocytes by 2-NBDG compared to the middle population. Supplementary Table S1. Normalized gRNA distributions for preadipocyte (P), adipocyte (A), Top 5% 2-NBDG staining (T), Middle 2-NBDG staining (M) and Bottom 5% 2-NBDG staining (B), as well as associated *p*-values and false discovery rates (FDR) for comparisons between groups for all gRNAs detected in this screen. Supplementary Table S2. Differential abundance of proteins of Rfwd2 Knockout Adipocytes. For proteins that had 50% valid values, we imputed the remaining missing values using random draws from a truncated distribution with parameters estimated using quantile regression and normalized the values such that all samples had the same median log intensity of proteins. Differential expression was tested with the limma R package. Supplementary Table S3. Loading values for Principal Component 1 in Figure 5B.
