# Supplementary figures and images for: A CRISPR Screen Identifies the E3 Ubiquitin Ligase Rfwd2 as a Negative Regulator of Glucose Uptake in Brown Adipocytes
Source: Genes (Basel). 2023 Sep 26;14(10):1865. doi: 10.3390/genes14101865 (PMC10606202; doi:10.3390/genes14101865)

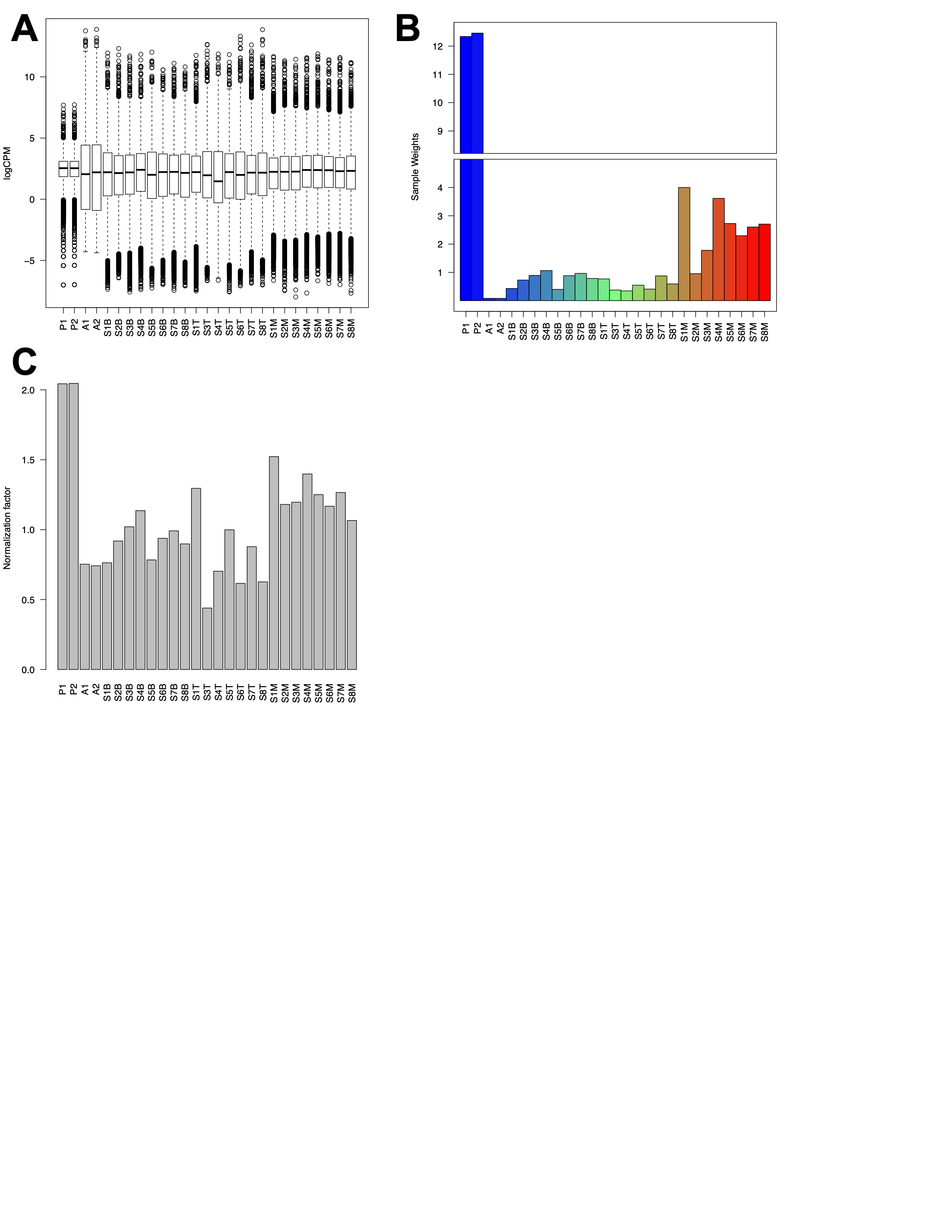

Supplement: Supplementary file 1 [file genes-14-01865-s001.zip › CRISPRbrownads_figuresv7.2SuppFig1.tiff]

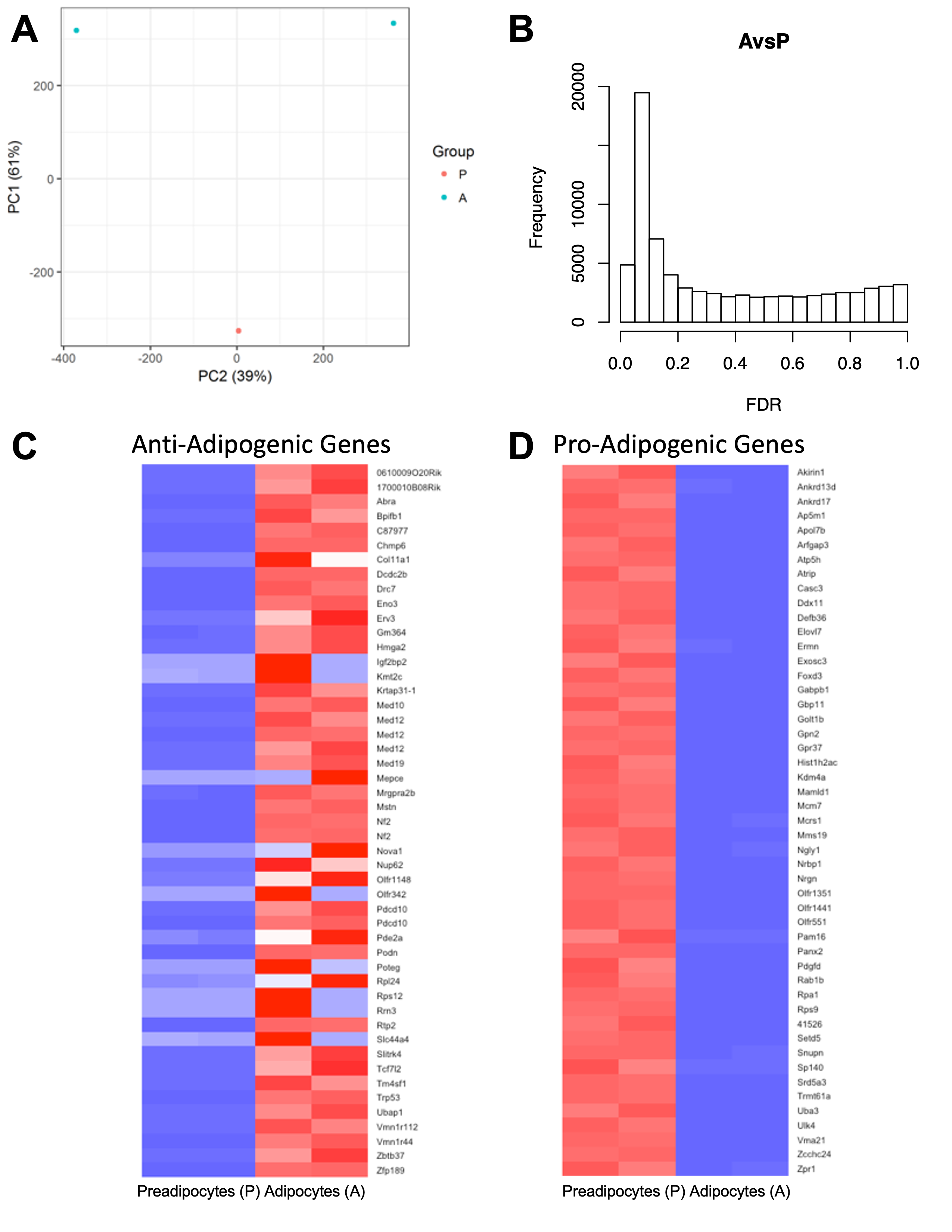

Supplement: Supplementary file 1 [file genes-14-01865-s001.zip › CRISPRbrownads_figuresv7.2SuppFig2.tiff]

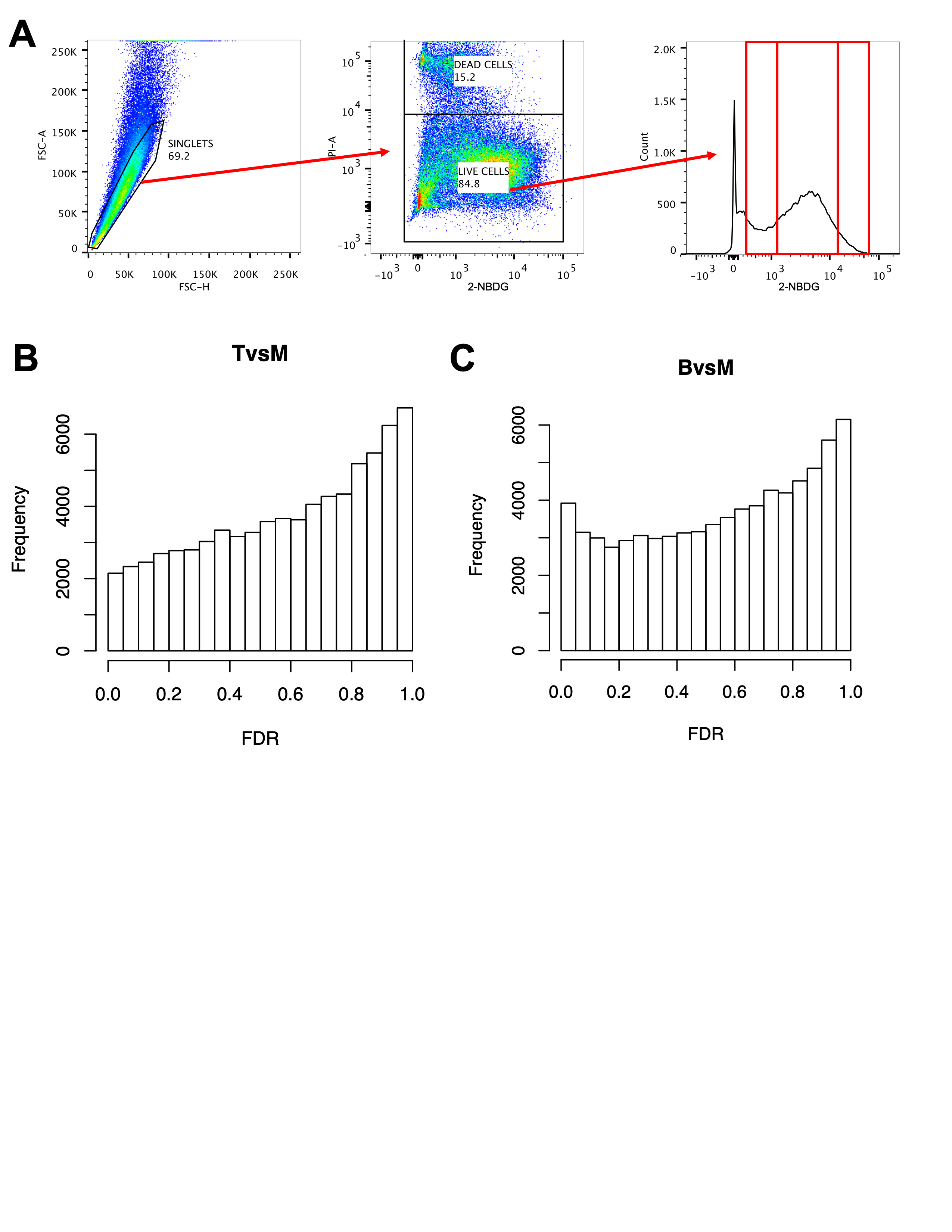

Supplement: Supplementary file 1 [file genes-14-01865-s001.zip › CRISPRbrownads_figuresv7.2SuppFig3.tiff]
